# Financing elderly people's long‐term care needs: Evidence from China

**DOI:** 10.1002/hpm.2488

**Published:** 2018-01-12

**Authors:** Fengyue Li, Junko Otani

**Affiliations:** ^1^ College of Marine Culture and Law Shanghai Ocean University Shanghai China; ^2^ Graduate School of Human Sciences Osaka University Osaka Japan

**Keywords:** China, disability, financing, long‐term care, long‐term care insurance

## Abstract

Confronted by accelerated population aging, China is establishing a long‐term care (LTC) system. This study discusses challenges and recommendations for financing China's LTC system. On the basis of the data on elderly people's self‐care ability from the Chinese Longitudinal Healthy Longevity Survey, we calculate the size of the elderly population that need LTC for the period from 2015 to 2030 and analyse the increasing tendency of LTC expenses by considering the impact of price increase. We also analyse the local governments' financial capacity for LTC support by comparing the expense level to the fiscal revenue. The study found that aging will double the LTC expenses by 2030. Therefore, this study suggests the establishment of an LTC insurance system that allocates LTC expenses, which are currently borne by individuals and families, more fairly among the government, individuals, and families. Moreover, with the current LTC reforms, implemented primarily by local governments in China, we believe that the central government should bear some of the fiscal responsibility by conducting fiscal transfers to partially support undeveloped regions that are establishing an LTC system.

## 1 INTRODUCTION

Population aging has become a serious issue in China. Recent census data indicate that people aged 65 years or older accounted for 119 million (8.9% of China's total population) in 2010 and it is projected to triple to 371 million by 2050. The rapid aging of the population has brought about social and economic impacts, including reduced labour supply and increased financial burden on the tax base, social security, and people's personal savings. Additionally, as the social care service system is still not comprehensive, the burden of care on families is getting heavier. Given the size of its population and the growth of its elderly members, China faces a major challenge in addressing the long‐term care (LTC) needs of its demography. The central government has issued a series of policies and measures in recent years, an elderly care system that offers a comprehensive range of services in both rural and urban areas, which is financially supported by individuals, families, communities, and care institutions. In addition, the government is gradually establishing a LTC insurance (LTCI) system. In September 2013, the State Council successively issued Decree Nos. 35 and 40 to propose the development of health insurance products and commercial LTCIs for old people.^1^, ^2^ In August 2014, the *Opinions on Acceleration of Modern Insurance Service Industry* proposed the development of commercial health insurance products linked with basic medical insurances and the development of commercial LTCI.^3^ In October 2015, the exploration of the establishment of the LTCI system and the implementation of LTCI pilots were written into the 13th Five‐Year Plan by the Fifth Plenary Session of the 18th Central Committee of the Communist Party of China.^4^ In July 2016, the Ministry of Human Resources and Social Security of the People's Republic of China issued the *Instructions on Carrying out Long‐term Care Insurance System Pilots*, which proposed that “the exploration of the establishment of LTCI system is a strategic measure for dealing with the aging tendency of the population and improving social and economic development; it is a significant livelihood project for sharing development and reform achievements and an important institutional arrangement for improving the social security system.”^5^ Fifteen cities were selected as pilot areas in the document, allowing the gradual exploration and establishment of a multichannel funding mechanism for LTCI with mutual assistance and responsibility.

China's distinct approach to social policy formulation often involves a number of pilots in pioneering cities before the central authorities incorporate successful experiences into the national policy framework.^6^ In recent years, local governments have implemented a variety of pilot policies. For example, in 2012, in the Shandong Province, the city of Qingdao took the lead by establishing LTCI, which covers the assured persons of medical insurance; the funds come from surplus funds of the medical insurance, so it does not require individual or employer contributions (or premiums). In July 2016, Beijing implemented its pilot LTCI system in the Haidian district by issuing the *Measures of Haidian District on Home‐based Care for the Aged and Disabled Mutual Insurance Pilot*,^7^, ^8^ which is a voluntary commercial insurance operated by insurance companies and established through government's policy and financial support. It is funded by a combination of individual contributions, government subsidies, and mutual funds paid by nursing service agencies. Meanwhile, Beijing proposed a policy‐type pilot LTCI (2017) in the Shijingshan District^9^; some provinces, including municipalities and autonomous regions, such as Shanghai and the Jiangsu Province also listed LTCI as a key task for 2016.^10^, ^11^

The various insurance models in pilot policies that have been implemented all over China have substantial differences in terms of financing; for example, Qingdao Jiangsu and Changchun drew from the surplus of the current medical health care insurance fund, while Shanghai used financial subsidies and individual contributions. Fund collection is one of the most important research topics, in deciding upon a future LTCI model for China. This study discusses challenges and recommendations for financing LTC in China. We forecast the LTC needs of elderly people for the coming 20 years on the basis of the elderly people's self‐care ability and population variation trends. We also calculate the increasing tendency of future LTC expenses for the same period and analyse the government's ability to financially support the demographic and health changes. In the final section of this paper, we suggest 2 points for reform and future directions for the LTC financing system in China.

## 2 MATERIALS AND METHODS

In China, LTCI is a new concept that has not yet been formally implemented in most provinces and cities. Moreover, it lacks classifications for different levels of care. As there are no official statistical data on LTC expenditure, scholars rarely perform empirical studies on LTC financing. However, many have provided policy suggestions for establishing an LTCI system in China.^12^, ^13^, ^14^ Due to this lack of official statistical data on LTC expenditure and certification standards for the LTC required in China, empirical studies on LTC demand are mainly divided into 2 classes: those that use data such as demographics and disability rates to quantify LTC needs and costs^15^, ^16^, ^17^, ^18^ and those based on field investigation data of individual cities and communities for analysing the tendency of care service needs.^19^, ^20^, ^21^, ^22^, ^23^ Studies that use data such as demographics and disability rate have discussed the general demand and the current and projected costs for China. At present, 2 methods—macrocosmic simulation and prediction and microcosmic simulation and prediction—are mainly used to predict the LTC demands of early people. Although the accuracy of microcosmic prediction is high, owing to multiple datasets and definitions of physical disabilities, research calculation results have large differences. In addition, for the estimation of the health matrix of transition probability, the estimation results of developed countries were also frequently used, which affected the accuracy of the estimation. This study used macrocosmic prediction to calculate the population of elderly people who need care according to changes in disability rates. It used data from the Beijing University China Health and Retirement Longitudinal Study (CHARLS)^24^ to calculate the distribution of elderly people's self‐care ability during the base period and consulted the microanalysis results of previous studies for elderly people's health changes. To predict the nursing expenses of elderly people, the study calculated the care costs according to various care levels.

The cost of LTC is closely related to self‐care ability. The most commonly used evaluation framework is activities of daily life (ADL), which are basic daily living activities that reveal self‐care ability. In China, the most commonly used ADL measure is the Katz Index of Independence in ADL, which was proposed by Katz in 1963, and it includes bathing, dressing, going to the toilet, transferring, feeding, and continence. Estimations of ADL in this study are based on the data from the CHARLS,^24^ which analysed 6 types of data about the daily lives of 6338 people who were 65 years or older in 2015; this sample was selected from a bigger sample of 21 095 subjects. There is no official classification for the different levels of self‐care status in China, so this study refers to the definitions by the Research Group of the China Research Center on Aging,^25^ which classifies elderly people who cannot take care of themselves as having mild disability (1‐2 ADL loss), moderate disability (3‐4 ADL loss), and severe disability (5 or more ADL loss).

To calculate the long‐run LTC needs, it is necessary to consider the changes in elderly people's ability to take care of themselves. There are 3 theoretical assumptions that relate to the potential disabilities of an aging person. The first is the “compression of morbidity,”^26^ which indicates that the number of years lived with no disability increases with changes in lifestyle. The second is the “expansion of morbidity,”^27^ which indicates that the level of medical care and disease prevention will prolong the life of a diseased population and the number of years lived with disability increases. The third is the “dynamic equilibrium,”^28^ which indicates that the proportion of life span with serious illness or disability stabilises or decreases, whereas the proportion with moderate disability or less severe illness increases.^29^ They have been studied by scholars on the changes in elderly adults' disability levels in China. Gu and Zeng^15^ reported that the proportion of elderly people living with disabilities declined by 1% per year from 1992 to 2002. Jiang and Wei's^30^ research and analysis of the health conditions of elderly people for the period from 2002 to 2011 also verified the compression of morbidity. However, Du and Wu^31^ reported that from 1994 to 2004, the proportion of elderly Chinese disability ratio increased by 18% from 7.5% to 8.9%. Huang^32^ assumed that the disability ratio in all ages remained unchanged. Such variation in the results and conclusions of existing studies may be due to the use of different sample data and indexes for health conditions, life expectancy, and so on. Based on the research described above, this study uses 3 statistical categories: low range, medium range, and high range possibilities. Low range assumes that the proportion of elderly people receiving LTC decreases at a rate of 1% per year, medium range assumes that this proportion does not change, and high range assumes that it increases at a rate of 1% per year. The need for LTC derives from society's demographics, especially the size and age composition of the population. Demographic census and projections give us a benchmark for estimating how many people will require care. This study forecasts elderly people's care needs for the period from 2015 to 2030 using China's census data^33^ and United Nations' population projections.^34^

## 3 RESULTS

### 3.1 The changing trend of LTC needs

According to the above definitions by the Research Group of the China Research Center on Aging^25^ and the CHARLS data, the self‐care ability of the elderly people was calculated for 2015. Table 1 indicates that the proportion of elderly people who cannot take care of themselves represented 12.8% of the total respondents and those having mild, moderate, and severe disabilities represented 9.1%, 2.2%, and 1.4%, respectively. Thus, in China, the total number of elderly people who needed care was 16.69 million in 2015, including 11.96 million, 2.89 million, and 1.84 million with mild, moderate, and severe disabilities, respectively.

**Table 1:** Health levels of old people in China (2015)

Age Group, y	Healthy, %	With Disability, %		
		Mild	Moderate	Severe
65‐74	90.5	7.2	1.4	1.0
75‐84	82.1	11.0	3.0	1.9
85+	68.1	19.3	8.5	4.1
65+	87.2	9.1	2.2	1.4

The results in Table 2 indicate that in the low range category, the annual growth rate of elderly people with disability is 3.1% and their LTC needs will increase from 16.69 million in 2015 to 26.56 million in 2030 (60% increase); in the medium range category, the annual growth rate is 4.2% and their LTC needs will increase to 30.88 million in 2030 (85% increase); and in the high range category, their LTC needs will increase to 35.85 million in 2030 (115% increase).

**Table 2:** Projection of China's long‐term care needs (in million)

Statistical Categories	Disability Level	2015	2020	2025	2030
Low range	Mild	11.96	14.68	16.51	19.03
	Moderate	2.89	3.55	3.99	4.60
	Severe	1.84	2.26	2.54	2.93
	Total	16.69	20.48	23.04	26.56
Medium range	Mild	11.96	15.43	18.25	22.13
	Moderate	2.89	3.73	4.41	5.35
	Severe	1.84	2.37	2.81	3.40
	Total	16.69	21.54	25.48	30.88
High range	Mild	11.96	16.22	20.16	25.69
	Moderate	2.89	3.92	4.88	6.21
	Severe	1.84	2.50	3.10	3.95
	Total	16.69	22.64	28.14	35.85

Low range assumes that the proportion of elderly people receiving LTC decreases at a rate of 1% per year, medium range assumes that this proportion does not change, and high range assumes that it increases at a rate of 1% per year.

Owing to the rapid increase of the aged population, linear increases in elderly people's LTC needs can be found in 3 care categories (ie, the low range, medium range, and high range categories). This result indicates that regardless of the change in elderly people's overall self‐care ability, whether it remained unchanged or worsened, LTC costs for this population will increase owing to the inevitable aging of the population. Therefore, we have to be prepared to adjust and improve social security policies to cope with severe challenges caused by substantial increases in costs for elderly people's care needs in the future.

### 3.2 Forecasting financial burden of LTC

Care cost is closely related to the health status of elderly people and care patterns, including home care and institutional care. The number and proportion of elderly people receiving home care and institutional nursing services vary across countries. In China, due to the cultural expectations of “filial piety,” which the younger generation is obligated to fulfil, many Chinese elderly people prefer to live at home instead of moving into nursing homes.^35^ Thus, China's LTC is dominated by family‐oriented care and supplemented by institutional care that is provided by the public or private sector. Various local governments have proposed “9073” project and “9046” project according to their actual conditions. For example, Shanghai and the provinces of Jilin and Sichuan have an LTC system in which 90% of the elderly people depend on family support, 7% on community support, and 3% on institutional nursing. Meanwhile, Beijing and the provinces of Jiangsu and Guangdong have proposed a system in which 90% of the elderly people depend on family support and socialised services, 6% on government‐provided community support, and 4% on institutional nursing.

In accordance with China's goal of providing care for elderly people, this study designs 2 scenarios for elderly people who need to be cared for in the future: scenario 1, wherein elderly people with mild disability are cared for at home with the support of community care services and those with moderate and severe disabilities receive services in institutional settings with the support of community care services, and scenario 2, wherein elderly people with mild disability and of lower age (65‐74) with moderate disability are cared for at home with the support of community care services and of higher age (over 75) with moderate to severe disability receive services in institutional settings. By investigating the per capita expenditure of elderly people for different levels of home and institutional care, we can calculate the total care cost for the base year by multiplying the average cost by the number of elderly people who are in need of care in the base year. Considering that the cost changes with the development of the national economy, it will be affected by factors such as economic growth, rising wages, and inflation. Therefore, we use the past price levels in health care services to calculate the future growth of care cost.

We investigated the institutional care expenses and home care subsidies of Qingdao because it was the easiest to implement a pilot LTCI there and use its care expense level as a good reference. According to our surveys, the standard charges with respect to care level and facilities in public care institutions are generally between RMB 1300 and 1600 per month and those in private care institutions are generally between RMB 1900 and 2600 per month. The average expense on care institutions in Qingdao is estimated to be RMB 1500 per month for moderate care and RMB 2000 per month for complete care. The government provides subsidies for LTC services at home for elderly people in Qingdao on 60 hours per month for those with complete disability and 45 hours per month for those with partial disability; the subsidies are given as per the standard charges of RMB 15 per hour in urban areas and RMB 10 per hour in rural areas. According to the above figures, the average cost subsidy for home care is estimated at RMB 750 per month for disabled elders and RMB 560 per month for partially disabled elders.

Through the investigation on the price of care services in the city of Qingdao, this study calculated the average price of LTC services in the whole country with the Consumer Price Index (CPI) in Qingdao and in the whole country. Using the same method to calculate the care price in Beijing and Shanghai and further investigate the actual standard charges, the study verified that the calculated results conform to the local conditions.

If care services are provided as per scenario 1 in Table 3, the total LTC costs will increase from RMB 176.6 billion in 2015 to RMB 325.3 billion in the low range category, to RMB 378.2 billion in the medium range category, and to RMB 439.1 billion in the high range category in 2030. If care services are provided as per scenario 2, the total nursing expenses will increase from RMB 169.6 billion in 2015 to RMB 316.6 billion in the low range category, to RMB 368.1 billion in the medium range category, and to RMB 427.4 billion in the high range category in 2030.

**Table 3:** Long‐term care cost changes in China

		(In Billion RMB)			
Scenario	Statistical Categories	2015	2020	2025	2030
1	Low range	176.6	227.5	268.7	325.3
	Medium range		239.3	301.5	378.2
	High range		251.5	328.3	439.1
2	Low range	169.6	214.4	255.7	316.6
	Medium range		225.5	282.7	368.1
	High range		237.0	312.3	427.4

Scenario 1: Elderly people with mild disability are cared for at home and those with moderate and severe disability receive services in institutional settings with the support of community care services. Scenario 2: Elderly people with mild disability and those of lower age (65‐74) with moderate disability are cared for at home with the support of community care services and those of higher age (over 75) with moderate to severe disability receive services in institutional settings.

In 2015, LTC cost was equivalent to 1.0% of the total public financial expenditure and 0.26% of the Gross Domestic Product (GDP). On an average, across OECD (Organisation for Economic Co‐operation and Development) countries, LTC cost was 1.6% of the GDP.^36^ Although China's expenditure levels are currently low as compared to the OECD countries, the changing demographic trends are such that the impact is greater than the OECD average. Thus, the need for LTC in China will increase at a rate that exceeds that of developed countries.

## 4 DISCUSSION

From the analysis in the previous subsection, we conclude that rapid population aging increases LTC cost significantly in both scenarios 1 and 2. Therefore, we need to analyse individual and government's abilities to financially support these demographic and health changes.

As regards the individual ability to cope with LTC needs, in China, the LTC system is in its early stages, the elderly people there are mainly cared for daily by family, which represent an informal care system. At present, the public financial support is only for partial nursing institutions and community health service; thus, the burden of expenses is borne by individuals and families. However, the average pension is only RMB 2373 per month in urban areas in 2016, which indicates that more than half of the elderly people in urban areas who need LTC cannot afford the cost of institutional care.

As regards the government's financial ability, we mainly discuss the provincial government's financial support capacity. In China, basic endowment insurance is paid and managed in provinces, while medical insurance is managed and used in municipalities. In recent years, the central government through transfer payments is enhancing the local governments' financial independence and guaranteeing their capability to provide public services. The LTC cost and government revenues of provincial regions are presented in Table 4.

**Table 4:** Regional fiscal revenues, LTC expenses, and old population

Region	Government Revenue (In Billion RMB)	Per Capita Government Revenue, RMB	Age 65+ (% of Population)	LTC Costs (In Billion RMB)	LTC Costs (% of Government Revenue)
Beijing	235.4	12 002	8.71	4.9	2.08
Tianjin	106.9	8261	8.52	2.3	2.20
Hebei	133.1	1854	8.24	5.8	4.39
Shanxi	97.0	2715	7.58	2.7	2.81
Inner Mongolia	107.0	4331	7.56	2.6	2.39
Liaoning	200.5	4583	10.31	7.3	3.63
Jilin	60.2	2194	8.38	2.6	4.27
Heilongjiang	75.6	1972	8.32	3.5	4.64
Shanghai	287.3	12 483	10.12	8.5	2.97
Jiangsu	408.0	5187	10.89	15.2	3.73
Zhejiang	260.8	4793	9.34	11.2	4.30
Anhui	114.9	1932	10.18	6.3	5.48
Fujian	115.1	3121	7.89	4.5	3.91
Jiangxi	77.8	1746	7.60	3.3	4.28
Shandong	274.9	2870	9.84	13.2	4.81
Henan	138.1	1469	8.36	7.4	5.39
Hubei	101.1	1767	9.09	5.8	5.78
Hunan	108.1	1647	9.78	7.0	6.47
Guangdong	451.7	4331	6.75	14.2	3.15
Guangxi	77.2	1677	9.24	4.0	5.23
Hainan	27.1	3125	7.80	0.6	2.38
Chongqing	95.2	3301	11.56	4.1	4.28
Sichuan	156.2	1942	10.95	9.0	5.77
Guizhou	53.4	1536	8.57	2.3	4.26
Yunnan	87.1	1895	7.63	3.0	3.44
Tibet	3.7	1221	5.09	0.1	2.04
Shaanxi	95.8	2567	8.53	3.3	3.45
Gansu	35.4	1383	8.23	1.6	4.61
Qinghai	11.0	1959	6.30	0.3	2.91
Ningxia	15.4	2437	6.41	0.4	2.85
Xinjiang	50.1	2295	6.19	1.2	2.48

Abbreviation: LTC, long‐term care.

We find that the regions with a high proportion of LTC expense as a percentage of government revenue can be classified into 3 categories: (1) the regions with high proportions of elderly people (65 and over) and low per capita fiscal revenue, which include Anhui, Hunan, Hubei, Guangxi, and Sichuan; (2) the regions with low proportions of elderly people and low per capita fiscal revenue, which include Hebei, Jiangxi, Henan, Heilongjiang, Jilin, Guizhou, and Gansu; and (3) the regions with high proportions of elderly people and above average per capita fiscal revenue, which include Shandong, Zhejiang, and Chongqing. Table 4 indicates that fiscal revenue is not sufficient to support the aging population in many regions, especially those in the first and second categories, creating difficulty in bearing LTC expenses for their local governments. A region's fiscal revenue should generally be positively related to its GDP. Therefore, the economically underdeveloped regions facing significant financial pressure for LTC, such as central and western regions, are facing the serious problem of “getting old before getting rich.” Local governments are responsible for fiscal expenditure on social security. Such a social security fiscal system, the biggest issue of which involves variations in fiscal expenditure on social security caused by the local governments' different economic developments and consequent fiscal capacities.

The central government has encouraged various LTC pilot projects in different cities in China, but such projects were mostly initiated by local governments with their own funding sources.^17^ At present, the direct financial investment for care of elderly people mainly consists of 2 parts: capital support for institutions and subsidies for individuals. Capital support for institutions implies that the local governments give preferential policy treatments to private sector development of elder care facilities, such as tax exemptions, subsidies on new and existing beds, land allotment or leasing for new construction, and reduced utility rates^37^; subsidies refer to the support in availing services and paying for expenses of elderly people facing financial difficulties, both for institutional care and home care. In fact, the amount of subsidies varies substantially across regions, for example, standard subsidies per bed in newly established care institutions, with the highest subsidy at RMB 16 000 per new bed in Beijing and the lowest at RMB 1000 per new bed in some areas. In addition, there are variations in governmental subsidies for care services for the elderly people owing to variations in the financial resources of different areas. In some areas with low economic levels, the subsidy is RMB 50 per month, whereas in some areas with high economic levels, the subsidy can be as high as RMB 900 per month. Without the financial support from the central government, some local governments with low economic levels may not be able to financially support their LTC system, a social security system that depends tremendously on such support. Therefore, channels for sustainable capital sources and explicit fiscal responsibilities of the governments at all levels must be explored and discussed.

## 5 CONCLUSION

With the rapid aging of its population, China faces the major challenge of addressing the LTC needs of its elderly people. A WHO study pointed out that the need for LTC services in developing countries such as China is increasing at a rate that exceeds the experience of developed countries.^38^, ^39^ A rapidly aging population, coupled with “getting old before getting rich,” has profound implications on China. The narrow financing base and vast population has resulted in a large unmet demand, calling for financing reforms.^6^ Until now, the elderly care costs are borne mostly by individuals and families, and with the government's envisioned new forms of LTCI and subsequent reforms, demand for LTC is expected to increase significantly. However, 2 important financing issues come with the establishment of a new LTC policy: division of intergovernmental fiscal responsibility and share of the burden of private payments.

At present, in China, tentative LTC reforms have been made primarily by local governments, but we argue that without the central government's support, China will not be able to establish a sustainable LTC system. If LTC systems integrated by local governments are implemented, intergovernmental transfer payments are needed to support the economically underdeveloped regions to enhance local governments' financial capacities and reduce regional differences.

There are guiding opinions on the implementation of LTCI system pilots proposed by the Ministry of Human Resources and the Social Security of the People's Republic of China. In terms of fund procurement, such opinions clearly specify that “in pilot phases, funds can be raised by optimising the overall account structures of employees' medical insurance, transferring the consolidated fund surplus of employees' medical insurance, and adjusting the rates of employees' medical insurance.”^4^ At present, Qingdao, which has implemented its LTC medical insurance, has realised the operation of a pilot system with the support of governmental financial funds by transferring (from medical insurance to LTC) the overall fund surplus of the Basic Medical Insurance for Employees and the Basic Medical Insurance for Urban/Rural Residents. However, the medical benefits fund operation of other areas is not so promising. By 2013, the revenues generated by employees' medical insurance funds of 225 areas in China were failing to meet their expenditures and 22 of them have spent the accumulative surplus of previous years; in terms of medical insurance for residents, the revenues in 108 areas were failing to meet the expenditures. According to the prediction in the 2014 Chinese Medical and Health Development Report, by 2017, the revenues of the basic medical insurance funds of employees in urban areas will fail to meet the expenditures, and by 2024, the accumulated surplus of such funds will have a significant deficit of RMB 735.3 billion.^40^ These projections indicate that from a long‐term perspective, a reasonable individual payment mechanism is necessary for the establishment of care insurance systems. Therefore, through the implementation of an LTCI system like that in other countries such as Japan, we can consider a mode of financing that combines the government's tax base with a personal social insurance fee. In the design of China's LTCI system, the research on LTC needs and financial implications is complex because multiple factors must be taken into consideration, including the future of the elderly population, the disability ratio, wage levels and retirement, the cost of different care conditions, social insurance payments and government financial burden, and the future sustainability of the LTC system. Moreover, the experience of other countries is useful in China's decision on whether to institutionalise LTCI.

## ETHICS STATEMENT

This research proposal has been reviewed and approved by the Research Ethical Review Committee of Global Human Sciences Department of Graduate School of Human Sciences of Osaka University, Japan, in September 2016.
